# Supplementary material for: A Global Screen for Assembly State Changes of the Mitotic Proteome by SEC-SWATH-MS
Source: Cell Syst. 2020 Feb 26;10(2):133–155.e6. doi: 10.1016/j.cels.2020.01.001 (PMC7042714; doi:10.1016/j.cels.2020.01.001)

Q7Z4H7 | HAUS6_HUMAN | HAUS6 DGT6 FAM29A KIAA1574
Monomer MW [kDa]: 108.621 Monomer expected elution fraction: 39

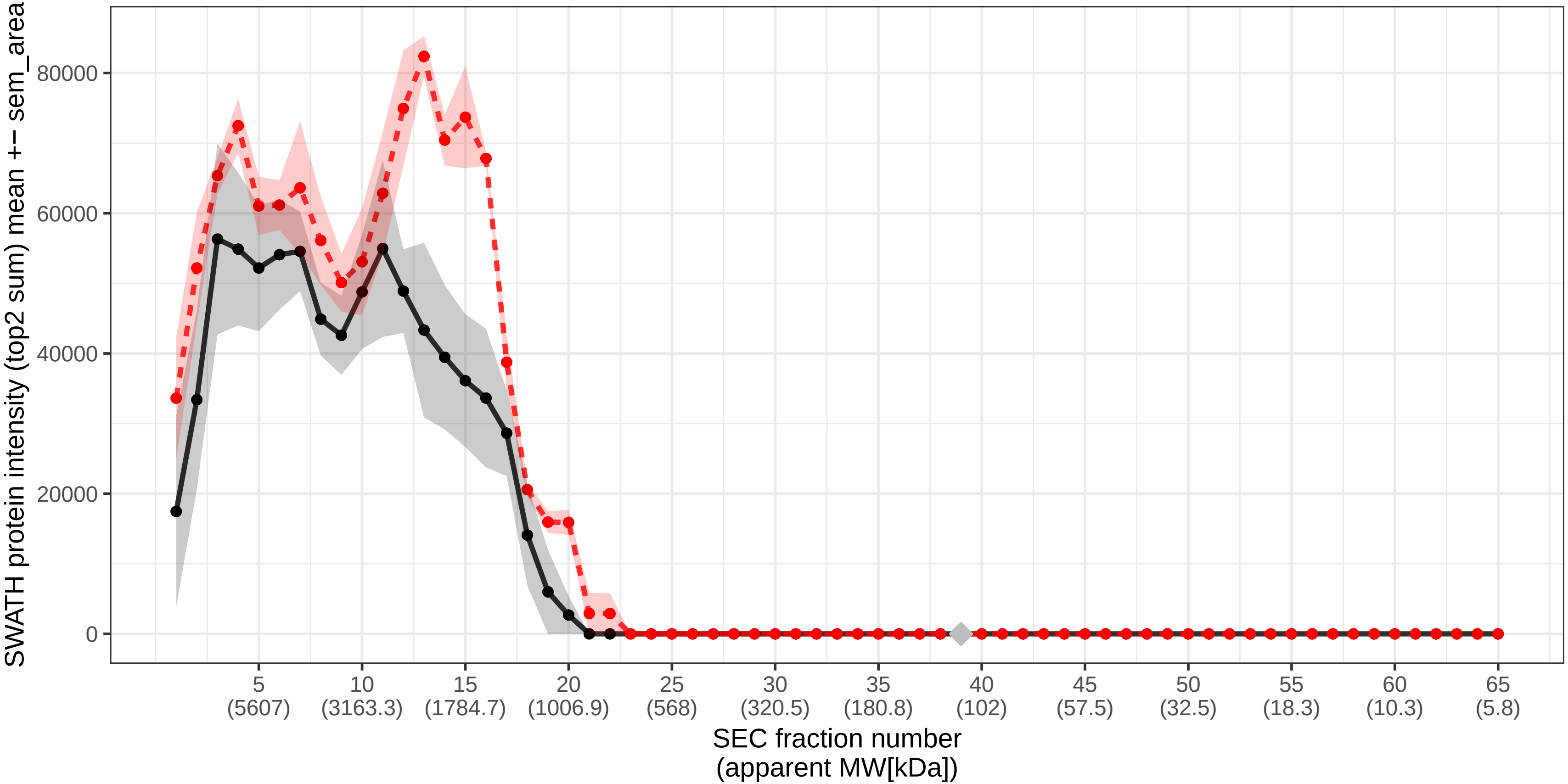

Supplement: Data S1. SEC-SWATH-MS Protein Chromatograms, Related to Figure 1 [file mmc6.zip › SECchrom_Q7Z4H7_HAUS6_HUMAN_HAUS6_DGT6_FAM29A_KIAA1574.pdf]
